# Mycoplasma hominis meningitis in an extremely preterm newborn: a case report

**DOI:** 10.1186/s12887-021-02532-3

**Published:** 2021-02-08

**Authors:** Najmus Sehr Ansari, Elizabeth Asztalos, Asaph Rolnitsky

**Affiliations:** Department of Neonatology Sunnybrook Health Sciences Centre, University of Toronto, 2075 Bayview Avenue, M Wing, M4N 3M5 Toronto, Ontario Canada

**Keywords:** Preterm neonate, Meningitis, Mycoplasma Hominis, Case report

## Abstract

**Background:**

Mycoplasma Hominis is a micro-organism which is a part of the human genitourinary tract flora. Neonates are susceptible to acquire this pathogen either in utero or through vertical transmission. In rare cases, it may cause central nervous system infections with severe morbidity and mortality in preterm and term neonates.

**Case presentation:**

We present a case of Mycoplasma Hominis meningitis in an extremely preterm neonate who presented with lethargy, tachycardia and seizures on day 7 of life. There was no history of maternal systemic or genitourinary infection during pregnancy and at the time of delivery. Empirical antibiotic therapy for neonatal meningitis was commenced after sending blood and cerebrospinal fluid cultures. Cerebrospinal fluid analysis showed pleocytosis with neutrophilic predominance, but no bacteria was identified on gram staining. Blood culture yielded no growth of any bacterial pathogen. However, growth of Mycoplasma Hominis was suspected in cerebrospinal fluid culture which was confirmed by 16S ribosomal ribonucleic acid (RNA) polymerase chain reaction analysis. Subsequently, antibiotics were changed to Moxifloxacin and Doxycycline which were given for a total duration of 6 weeks. Multiple cerebrospinal fluid cultures were performed during this treatment. No growth of any pathogen was identified on any of these cerebrospinal fluid cultures.

**Conclusions:**

We report a rare case of Mycoplasma Hominis meningitis in an extremely preterm neonate which was successfully treated with a combination therapy of Moxifloxacin and Doxycycline.

It is important to consider the possibility of Mycoplasma Hominis meningitis in neonates who present with clinical signs and pleocytosis in cerebrospinal fluid but negative gram staining and no growth on conventional culture media.

## Background

Mycoplasma Hominis is a frequent habitant of the human genital tract [1]. Neonates are susceptible to acquire this microbe either in utero or through the colonized birth canal during the process of parturition [2]. However, invasive infections with this organism in preterm and term infants are noted to be rare [1, 3]. We describe an unusual case of neonatal meningitis due to Mycoplasma Hominis, confirmed in bacterial cultures and 16s ribosomal RNA Polymerase chain reaction (PCR) analysis. This case report demonstrates the importance of suspecting Mycoplasma Hominis as a cause of central nervous system (CNS) infection in neonates who present with clinical signs but no growth on conventional bacterial cultures and no improvement on empirical antibiotic treatment.

## Case presentation

A male neonate was born at 25 + 6 weeks of gestation to 35 years old gravida 2, parity1 (G2P1) mother with antiphospholipid antibody syndrome. This was an in vitro fertilization (IVF) pregnancy. She was Rubella immune and negative for Hepatitis B surface antigen, Venereal disease research laboratory test (VDRL), Human immunodeficiency virus, Gonococci and Chlamydia. Her urine culture and vaginal swab for Group B Streptococci was also negative. Her anatomy scan at 18 weeks of gestation showed normal fetal anatomy and a short cervix for which she underwent cervical cerclage placement at 20 + 5 weeks gestation. There was no history of chorioamnionitis or prolonged rupture of membranes. She presented at 25 + 5 weeks with preterm labor and had a spontaneous vaginal delivery the next day. She had received two doses of Betamethasone and Magnesium sulphate prior to delivery. He initially required positive pressure ventilation followed by intubation and surfactant administration with an acceptable response. Umbilical arterial and venous catheters were placed and the neonate was transferred to neonatal intensive care unit (NICU) in a stable condition.

A blood culture was drawn at birth and empiric treatment with Ampicillin and Gentamicin was intiated. The antibiotics were discontinued after 36 hours as the blood culture showed no growth and he remained clinically stable. In addition, as per unit protocol, an endotracheal aspirate was sent for a Ureaplasma culture on admission. This grew a Mycoplasma species for which 3 days of intravenous Azithromycin was given.

A head ultrasound on day 4 of life showed bilateral intraventricular hemorrhage (grade 3) with mild ventriculomegaly and a hemorrhagic venous infarct in the right frontoparietal region. Umbilical arterial catheter was removed on day 4 of life whereas umbilical venous catheter was removed on day 6 of life. He remained stable till day 7 of life when he presented with an acute clinical deterioration presenting as lethargy and tachycardia; a septic work up including blood and cerebrospinal fluid (CSF) cultures was completed and Vancomycin and Cefotaxime were started as empiric antibiotics. His CSF analysis showed red blood cells (RBC) count of 4887 × 10E6/l, pleocytosis of white blood cells (WBC) 9620 × 10E6/l with neutrophilic predominance, hyperproteorachia - protein 8300 mg/l and hypoglycorachia - glucose 0.1 mmoles/liter. Gram staining in CSF was negative. Within hours he developed generalized clinical seizures which was treated with Lorazepam and Phenobarbital followed by a Levetiracetam load and maintenance dose. He had further episodes of clinical and subclinical seizures which resolved after further adjustment of levetiracetam doses.

His first CSF culture was suspected to grow Mycoplasma Hominis and was sent to the regional national diagnostic laboratory for confirmation. The CSF culture was repeated along with a sample for 16s ribosomal RNA PCR analyses. The 16s ribosomal RNA PCR analysis confirmed Mycoplasma hominis in both CSF samples. Antibiotics were then changed to Moxifloxacin and Doxycycline. The repeat CSF culture after 48 hours of Moxifloxacin and Doxycycline showed no growth. CSF analysis done seven days after starting this treatment showed RBC 5650 × 10E6/l, WBC 449 × 10E6/l, protein 3763 mg/l and glucose 0.2 mmoles/liter. Subsequent CSF findings repeated after another three days showed further improvement, RBC 7187 × 10E6/l, WBC 93 × 10E6/l, protein 3691 mg/l and glucose 0.5 mmoles/liter. No growth was seen on any of these CSF cultures. Serial head ultrasound scans were done which showed progressive ventriculomegaly, post hemorrhagic ventricular dilatation and cystic evolution of hemorrhagic/venous infarct in the right frontoparietal region. Although these findings could be attributed to extreme prematurity; there is a likelihood that infection with Mycoplasma Hominis may have played a role in its progression. There have been documented cases of Mycoplasma Hominis meningitis resulting in CNS complications including intraventricular and periventricular hemorrhage, hydrocephalus, and infarction [3]. Lumbar taps were repeated as therapeutic measure to reduce ventriculomegaly, but optimal volumes of CSF were not obtained. Because of the ventriculomegaly, he was evaluated for a possible shunt on day 31; this was deferred because of the stable ventriculomegaly.

Antibiotics were given for a total duration of six weeks after which he was discharged home with a normal neurological examination. He was referred to Neurodevelopmental clinic for follow-up.

## Discussion and conclusion

Neonatal CNS infections with Mycoplasma Hominis, although rare, can cause severe morbidity and mortality in neonates [1]. In a case series of 29 neonates with Mycoplasma Hominis infection (age of presentation day1-32 of life), Hata and colleagues reported complications such as brain abscess, hydrocephalus, infarction, cerebritis and periventricular/intraventricular hemorrhage in 34 % cases, death in 28 % and sequelae mostly hemiparesis in another 28 % cases [3]. This may be attributed to a delay in diagnosis, ineffective antibiotic treatment, or suboptimal treatment regimens for neonatal CNS disease [3, 4]. Therefore, prompt diagnosis, early initiation and optimal duration of appropriate antibiotic therapy is necessary for a favorable prognosis.

The clinical presentation may include apnea, temperature instability, lethargy, vomiting, irritability poor tone, twitching or seizures [7]. Detection of Mycoplasma Hominis can be challenging since they lack peptidoglycan cell wall which renders them unidentifiable by gram staining [5]. In addition to this, they grow very slowly on routine culture media and require a specific blood agar medium for their detection [1, 6]. Due to this, there is a likelihood that Mycoplasma Hominis infections may remain undiagnosed or diagnosed late in infants presenting with clinical signs and symptoms [2]. Hence, it is important to consider the possibility of Mycoplasma Hominis infection in cases where the CSF shows pleocytosis and no growth of organism on routine culture media. A 16S ribosomal RNA PCR analysis has proven useful for detection of Mycoplasma Hominis in blood and CSF which are difficult to grow on standard culture media [7, 8]. The microbe is identified by direct sequencing analysis after amplification by PCR. Use of pathogen-specific primers in 16S RNA analysis results in rapid detection of the specific organism [4]. Prematurity, low birth weight, and neural tube defects are recognized to be the most common risk factors for neonatal meningitis with Mycoplasma Hominis [6]. However, it has also been seen in term neonates with no neurological birth malformations [1, 3].

The treatment options for Mycoplasma Hominis meningitis and its duration remains unclear [1, 9]. Due to the rarity of this infection in neonates, the current recommendations are based on clinical experiences and in-vitro susceptibility test results [9, 10].

Mycoplasma Hominis has shown susceptibility to Chloramphenicol, Tetracyclines, Lincosamide and Fluoroquinolones in in-vitro testing [11]. Fluoroquinolones have been used in past to treat neonatal mycoplasma hominins meningitis successfully [1, 3, 5]. Moxifloxacin, a fourth-generation fluoroquinolone is preferred because of its ability to concentrate in CSF and its bactericidal effects in CNS infections [1, 12, 13]. Although there are cases which were successfully treated with Moxifloxacin monotherapy [1, 6], there is a risk of development of resistance with fluoroquinolones during treatment [9, 14, 15]. Our literature search revealed only two reported cases of neonates who were given Moxifloxacin in combination with another antibiotic for Mycoplasma Hominis meningitis [3, 5] (Table 1).

**Table 1:** Characteristics of neonates treated with combination therapy (Moxifloxacin and another antibiotic) for Mycoplasma Hominis CNS infection

	Gestational Age	Age at presentation	Clinical presentation	Confirmation of diagnosis	Empirical antibiotic therapy and duration	Antibiotics for Mycoplasma Hominis	Outcome
Hata and colleagues^3^	38 weeks	25 days	Fever, vomiting, focal seizures	16S RNA PCR	Ampicillin & Cefotaxime (day 1–2) Ciprofloxacin (day 6–17) Acyclovir (day 6–8) Chloramphenicol (day 8–17)	Minocycline for 28 days (day 6–34) Moxifloxacin for 17 days (day 17–34)	Recovery with left hemiplegia
Watt and colleagues^5^	26 weeks	7 days	Apnea, hypotonia, generalized seizures	16S RNA PCR	Ampicillin and Gentamycin (day1-8) Ceftazidime (day 1–5 ) Vancomycin (day 8–19 ) Acyclovir (day 8–19) Meropenem (day 12–19 )	Doxycycline for 6 weeks (day 19–60) Moxifloxacin for 6 weeks (day 19–60)	Recovery, neurological outcomes not reported
Our case	25 + 6 weeks	7 days	Lethargy, tachycardia, generalized seizures	16S RNA PCR	Ampicillin and Gentamycin (day 1–2) Azithromycin (day 3–6) Vancomycin and cefotaxime (day 7–10)	Doxycycline for 6 weeks (day 10–52) Moxifloxacin for 6 weeks (day 10–52)	Recovery with normal neurological examination at discharge, follow up at 6 months showed normal neurological examination

Evidence from previously published cases show that 28 % neonates with Mycoplasma Hominis CNS infection died whereas 34 % had some CNS complications and 28 % cases developed some neurological sequelae mostly hemiparesis [3]. In our case, treatment with a combination therapy of Moxifloxacin and Doxycycline resulted in significant clinical and laboratory improvement in terms of negative CSF culture after 48 hours of initiating this regimen and decreased WBC count and protein in subsequent CSF analysis. Our patient was discharged home with a normal neurological examination and stable ventriculomegaly. His neurodevelopmental follow up at six months of age showed normal neurological findings.

It is important to consider Mycoplasma Hominis as a potential cause for neonatal meningitis in infants particularly those with previous colonization with this rare but devastating species. Our case demonstrates effective eradication of Mycoplasma Hominis with a combination therapy of Moxifloxacin and Doxycycline. However, further research is required to understand the pharmacokinetics of these antibiotics to establish optimal dosing and duration for effective treatment of CNS infections with Mycoplasma Hominis.

## Data Availability

Not applicable.

## Abbreviations

G2P1: Gravida2, Parity1
PCR: Polymerase chain reaction
RNA: Ribonucleic acid
IVF: In vitro fertilization
CSF: Cerebrospinal fluid
RBC: Red blood cells
WBC: White blood cells
CNS: Central nervous system
VDRL: Venereal disease research laboratory test
NICU: Neonatal intensive care unit

## References

[1] Wildenbeest JG, Said I, Jaeger B, van Hest RM, van de Beek D, Pajkrt D (2016). Neonate with Mycoplasma hominis meningoencephalitis given moxifloxacin. Lancet Infect Dis.

[2] Knausz M, Niederland T, Dósa E, Rozgonyi F (2002). Meningo-encephalitis in a neonate caused by maternal Mycoplasma hominis treated successfully with chloramphenicol. J Med Microbiol.

[3] Hata A, Honda Y, Asada K, Sasaki Y, Kenri T, Hata D (2008). Mycoplasma hominis meningitis in a neonate: case report and review. J Infect.

[4] Taku K, Hoshina T, Haro K (2017). An infant case with hydrocephalus as the initial manifestation of Mycoplasma hominis-associated meningitis. J Infect Chemother.

[5] Watt KM, Massaro MM, Smith B, Cohen-Wolkowiez M, Benjamin DK, Laughon MM (2012). Pharmacokinetics of moxifloxacin in an infant with Mycoplasma hominis meningitis. Pediatr Infect Dis J.

[6] Nohren J, Namtu K, Peloquin C, Messina A, Tuite G, Berman DM (2020). The pharmacokinetics of moxifloxacin in cerebrospinal fluid following intravenous administration: a report of successfully treated infant with mycoplasma hominis meningitis. Pediatr Infect Dis J.

[7] Woo PC, Lau SK, Teng JL, Tse H, Yuen KY (2008). Then and now: use of 16S rDNA gene sequencing for bacterial identification and discovery of novel bacteria in clinical microbiology laboratories. Clin Microbiol Infect..

[8] Mignard S, Flandrois JP (2006). 16S rRNA sequencing in routine bacterial identification: a 30-month experiment. J Microbiol Methods.

[9] Krausse R, Schubert S (2010). In-vitro activities of tetracyclines, macrolides, fluoroquinolones and clindamycin against Mycoplasma hominis and Ureaplasma ssp. isolated in Germany over 20 years. Clin Microbiol Infect.

[10] Waites KB, Katz B, Schelonka RL (2005). Mycoplasmas and ureaplasmas as neonatal pathogens. Clin Microbiol Rev.

[11] Watson L, Pang YM, Mitchell S, Dodgson A (2008). Mycoplasma hominis meningitis in a 24 week premature neonate: case report and short literature review. J Pediatr Pharmacol Ther.

[12] Alffenaar JW, van Altena R, Bökkerink HJ (2009). Pharmacokinetics of moxifloxacin in cerebrospinal fluid and plasma in patients with tuberculous meningitis. Clin Infect Dis.

[13] Di Paolo A, Gori G, Tascini C, Danesi R, Del Tacca M (2013). Clinical pharmacokinetics of antibacterials in cerebrospinal fluid. Clin Pharmacokinet.

[14] Bebear CM, Bové JM, Bebear C, Renaudin J (1997). Characterization of Mycoplasma hominis mutations involved in resistance to fluoroquinolones. Antimicrob Agents Chemother.

[15] Meng DY, Sun CJ, Yu JB, Ma J, Xue WC (2014). Molecular mechanism of fluoroquinolones resistance in Mycoplasma hominis clinical isolates. Braz J Microbiol.

